# An evaluation of the prevalence of physical health comorbidities in patients with severe and enduring mental illness following admission to the general adult psychiatric inpatient wards in Mersey Care NHS Foundation Trust

**DOI:** 10.1192/bjo.2021.261

**Published:** 2021-06-18

**Authors:** Declan Hyland, Millie Prime, Ellen Carter

**Affiliations:** 1 Consultant Psychiatrist, Clock View Hospital, Liverpool, Mersey Care NHS Foundation Trust; 2 5th year medical undergraduate, University of Liverpool

## Abstract

**Aims:**

This evaluation aimed to establish the prevalence of physical health comorbidities in SMI patients admitted to the general adult wards in Mersey Care NHS Foundation Trust.

**Background:**

Mean life expectancy in individuals with severe and enduring mental illness (SMI) is 15-20 years shorter than that of the general population. A significant proportion of excess mortality in patients with SMI is due to natural causes, e.g. cardiovascular disease and type II diabetes mellitus. Although SMI patients are at greater risk of developing chronic physical health problems, they often receive worse health care than the general population. SMI patients more likely to engage in unhealthy lifestyle behaviours, such as poor dietary choices, smoking and physical inactivity; Antipsychotic medication prescribed to these patients can cause adverse metabolic side effects.

**Method:**

A list of all inpatients on the eight general adult wards in the Trust was obtained in September 2020, producing a sample of 135 inpatients.

An audit tool was designed, capturing demographic data – gender, age, ethnicity, and also recording whether the patient had a diagnosis of an SMI (e.g. schizophrenia, bipolar affective disorder). The presence of any physical health comorbidities and whether the inpatient was a smoker was also recorded.

**Result:**

Of the 135 inpatients, 10 didn't have any physical health monitoring completed and were excluded from the sample, making the final sample 125 inpatients. 68 of the inpatients were male, 57 were female. 98 had a diagnosis of an SMI, 27 did not. Most inpatients were of “white British” ethnicity. Of the 98 SMI patients, 14 had type II diabetes mellitus, 11 had essential hypertension, 12 had chronic obstructive pulmonary disease and 22 were obese (i.e. a BMI > 30 kg/m2). 70 of the 98 patients with an SMI were smokers.

**Conclusion:**

As expected, a significant proportion of patients with SMI admitted to the general adult inpatient wards are smokers. Whilst admission to hospital may not be considered an ideal time to get patients to consider quitting smoking, admission does at least provide an opportunity to educate patients on the negative effects on physical health that smoking has. This evaluation has highlighted that physical health comorbidities are common in this patient group. Admission to the psychiatric ward provides a golden opportunity to provide education to patients on the importance of making healthy lifestyle choices and also to assess any physical health comorbidities and ensure the management of any such comorbidities is optimised prior to discharge.

